# Systematic analysis of the pharmacological content of the Tatort (scene of crime) TV series from 2019 to 2021

**DOI:** 10.1007/s00210-023-02427-3

**Published:** 2023-03-06

**Authors:** Marcel Borchert, Roland Seifert

**Affiliations:** Institute of Pharmacology, Hannover Medical School, Carl-Neuberg-Straße 1, 30625 Hannover, Germany

**Keywords:** Scene of crime, Therapy, Medication, Television, Public awareness

## Abstract

Tatort (scene of crime) is a must-see programme on Sunday evenings for many Germans. With its enormous reach, the crime series also deals in more than half of its episodes with active pharmacological substances, surprisingly most of them are used curatively. There are lots of ways representing the active pharmacological substances: simply naming the preparation to details such as information on how to take it or how it is manufactured illegally. Diseases of great interest to the public such as hypertension or depression are taken up. In addition to the correct presentation, in 20% of cases the active pharmacological substances were presented incorrectly or implausibly. But even with correct presentation, it may yield a harmful influence on viewers: Stigmatisation of preparations occurred in 14%, especially in the case of active pharmacological substances that are used in psychiatric therapies; presentations that could be considered dangerous occurred in 21% of the mentions. In 29%, content was presented positively to the audience beyond the correct presentation. Analgesics and active pharmacological substances used in psychiatry are frequently titled. But drugs such as amiodarone, insulin or "cortisone" are also mentioned. The potential for misuse is also presented. Tatort also educates the audience about common diseases and their treatment, for example hypertension, depression or the use of antibacterial drugs. However, the series does not educate the general public on the mechanism of action of commonly used drugs. There is a natural dilemma between informing the public and guiding them to misuse medicines. Finally, we discuss how future episodes could be improved with respect to pharmacological content.

## Introduction

With an average of 8.3 million viewers on Sundays at 8:15 p.m., the “Tatort” on the German television channel “Das Erste®” of the German joint organisation of regional public broadcasters as well as on the Austrian public broadcaster ORF® and on the Swiss public broadcaster SRF® is one of the most watched crime series on German television (https://de.statista.com/statistik/daten/studie/377327/umfrage/fernsehzuschauer-der-krimireihe-tatort/, last accessed on 03.11.2022). The first episode was broadcasted in 1970 (https://www.daserste.de/unterhaltung/krimi/tatort/kommissare/bildergalerie-ehemalige-kommissare-50-jahre-tatort100.html, last access on 25.01.2023) and is still running, making it the longest-running German TV drama. It is produced in German-speaking regions, with an average of 30 90-min episodes produced per year. Each episode typically features a closed plot, beginning with a violent crime, usually involving a corpse. Major cities in Germany as Munich, Berlin and Hamburg have their own teams of investigators with unique dialects, settings and detective personalities. In addition to Germany, “Tatort” is broadcasted in 50 other countries such as Poland, Iran and the USA, partly under the name “Scene of Crime”®. With this enormous reach, the series also casually informs about various pharmacological agents, some of which are mentioned as murder weapons, but also for curative therapies. Each TV station decides for itself (if necessary, in consultation with appropriate experts) in which way medical content is shown. In Germany, there is no higher authority for prior checking as Petra Putz, ARD®-Programme Directorate declared in an email dated May 19, 2022. In addition to the analysis of the individual active pharmacological substances and checking for plausibility in the presentation and use, there are possibilities of potential dangers through errors or imitation. Also, the question of what content is presented is important: from all this, errors in treatment by medical profession, positive and negative behaviour of normal viewers in dealing with drug therapies or also dangers in using active pharmacological substances and stigmatisation of them can emerge.

This situation prompted us to systematically analyse the pharmacological content of contemporary “Tatort” episodes from 2019 to 2021.

## Materials and methods

### Accessibility of the data

The episodes were retrospectively analysed over a 3-year period via the ARD media library or on the Amazon® channel “Das Erste Plus” in the original language in German. Included are all the episodes from 2019 to 2021 which were first broadcasted on ARD®. Due to lack of availability, the episodes “Murot and the marmot” (“Murot und das Murmeltier”), “The monster of Kassel” (“Das Monster von Kassel”), “Home sweet Home” (“Glück allein”) and “Meat loaf” (“Falscher Hase”) were excluded.

The “Tatort” is financed by the revenues of the GEZ (Gebühreneinzugszentrale der öffentlich-rechtlichen Rundfunkanstalten” (fee collection centre of the public service broadcasters in Germany)). GEZ collects a TV tax for public broadcasting in Germany, which amounts to 18.36€ per month for every household. In Germany, it is compulsory to pay this fee for news, sports, documentaries and entertainment. One can also find lots of content on the Internet. But with the provision of the 12th Amendment to the Interstate Broadcasting Treaty (“12. Rundfunkänderungsvertrag”), GEZ-financed content is only allowed temporarily on the Internet https://www.daserste.de/unterhaltung/krimi/tatort/videos/grenzfall-video-tgl-ab-20-uhr-100.html, last accessed on 03.11.2022). Three episodes, which were mentioned above, were no longer available for streaming.

### Statistical analysis

The active pharmacological substances mentioned in the movie franchise were extracted and categorised according to the given name within the episode, the purpose used, the presentation within the scene and the statements made in terms of content. In addition, it was assessed whether the presentation was plausible, whether important information was given to the viewers, whether there was a stigmatisation or heroisation of the substance, whether the use was poorly handled or whether a danger in the sense of possible misuse or risky procedures must be assumed.

The episodes were numbered according to their release. The order is based on the list found on (Wikipedia Germany, 2022) the free encyclopaedia on the Internet (https://de.wikipedia.org/wiki/Liste_der_Tatort-Folgen, last accessed on 17.11.2022).

## Results and discussion

### Use of active pharmacological substances

**Fig. 1:**
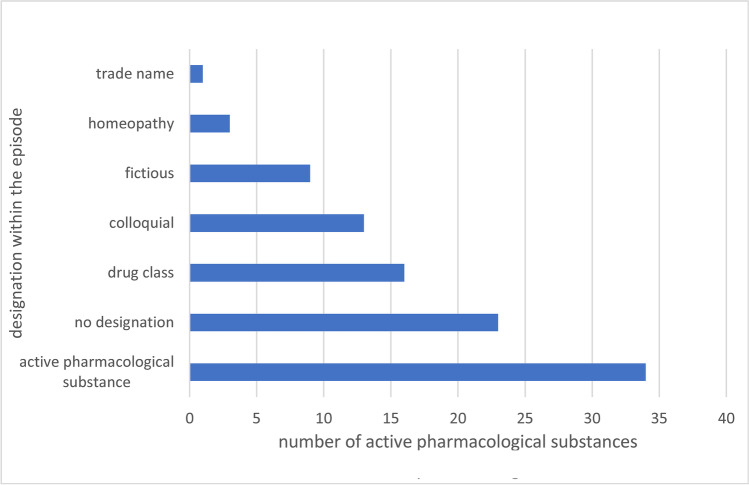
Designation of the active pharmacological substances. The designation of the active pharmacological substances within the Tatort episodes shown as a bar-chart, with values for the designation within the episodes

In total, 92 active pharmacological substances were named in 51 different episodes out of 101 episodes (Fig. 1). Analgesics, sedatives and anaesthetics were mentioned the most often (49%); for example, fentanyl and lorazepam were named directly. The second largest group were drugs used in internal medicine (24%), followed by psychiatric (excluding sedatives) (10%) medications, followed by herbal preparations (8%) and antidotes (7%). Homoeopathic remedies (3%) were the least common category. Table 1 shows an overview of the names given in TV episodes to active pharmacological substances. They were ordered by pharmacological groups.

**Table 1:** Pharmacologically active substances in “Tatort” from 2019 to 2021

Group	Active pharmacological substance/designation
Opiates/opioids	**Fentanyl** (3x), **Carfentanyl** (1x), **Morphine/opium/opioid** (3x)
Benzodiazepines	**Lorazepam** (2x), **Diazepam** (2x), **Benzodiazepines** (3x)
NMDA receptor antagonist	**Ketamine** (1)
Painkiller	**Ibuprofen** (2x), (head) pain tablet (6x)
Sedative	Syringe/medicine (7x), sleeping pill (4x)
Anaesthesia	**Chloroform** (4x), rape drugs (2x), general name (2x), local anaesthesia (2x), muscle relaxant (1x)
Psychiatric	**Ritalin**^**®**^(1x), “**antidepressant**” (5x), “**psychotropic drugs**” (3x)
Internistic (cardiovascular)	**Amiodarone** (1x), digitalis*(1x), **ephedrine** (1x), **antihypertensives** (2x), “against cardiac arrhythmia” (1x), “heart tablet” (1x)
Internal (others)	**Cortisone** (2x), **insulin** (2x), **G40/glucose** (2x), **magnesium** (1x), beta2-AR agonist (1x), **antibiotic** (2x), **anticoagulants (1x),** eye drops (1x), (probably) anticonceptive (1x), oxygen (1x)
Antidote	**Naloxone** (2x), **flumazenil** (1x), others (3x)
Herbal	**Vitamins** (3x), **caffeine** (2x), general (2x)
Homoeopathy	Alpha-latrotoxin** (1x), general (1x), red foxglove* (1x)

The table presents the active substances that appeared in the individual episodes in 2019–2021 and sorts them by pharmacological groups. The number in parentheses indicates the number of different episodes in which the active pharmacological substance is mentioned

Terms in bold designate specific active substances or preparations presented in “Tatort”

^*^The red foxglove is cut into a scene and could iconographically stand for the digitalis preparation. In homoeopathy, foxglove is used for sleep disorders

^**^Alpha-latroxin is a neurotoxin of the spider “black widow” and is used in homoeopathy for the treatment of hypertension

In 78 mentions (~ 73% of the total), active pharmacological substances were used for curative purposes. In only 20 mentions (~ 22%), they were used to kill or harm people. In ~ 40% of these cases, active substances were misused for stunning because of abductions, in ~ 60% for direct or indirect killing through, for example, withholding the correct medication. The remaining 10 mentions (~ 11%) used active pharmacological substances in the context of dependence syndromes. Multiple mentions occurred as well: active pharmacological substances were presented as curative within some episodes and were nevertheless misused.

Active pharmacological ingredients were particularly frequently titled with actual active ingredients (> 30%) (mostly in addition to other given names in the same episode); half of them were analgesics. Despite the official ban on product placement (according to Sven Döbler (MDR editor) in an email from May 31, 2022), Ritalin® (methylphenidate which is used for treatment of attention deficit hyperactivity disorder (ADHD)) was mentioned as a trade name in one episode. Fictitious product names were given in almost 10%; frequently for analgesics, although when the real active pharmacological ingredient mentioned. Drug classes were used primarily for psychiatric medications with mentions such as “antidepressants” (“Antidepressiva”) (episodes 1090, 1102, 1181) and “psychotropic drugs” (“Antipsychotika”) (episodes 1132, 1149), but also for medications used in internal medicine like “antibiotics” (“Antibiotika”) (in total 17%). In 14% of the cases, the active pharmacological substances were given in colloquial titles (such as “heart medication” (“Herzmedikation”), e.g., episode 1165), but also without designation in 25% of the cases.

**Fig. 2:**
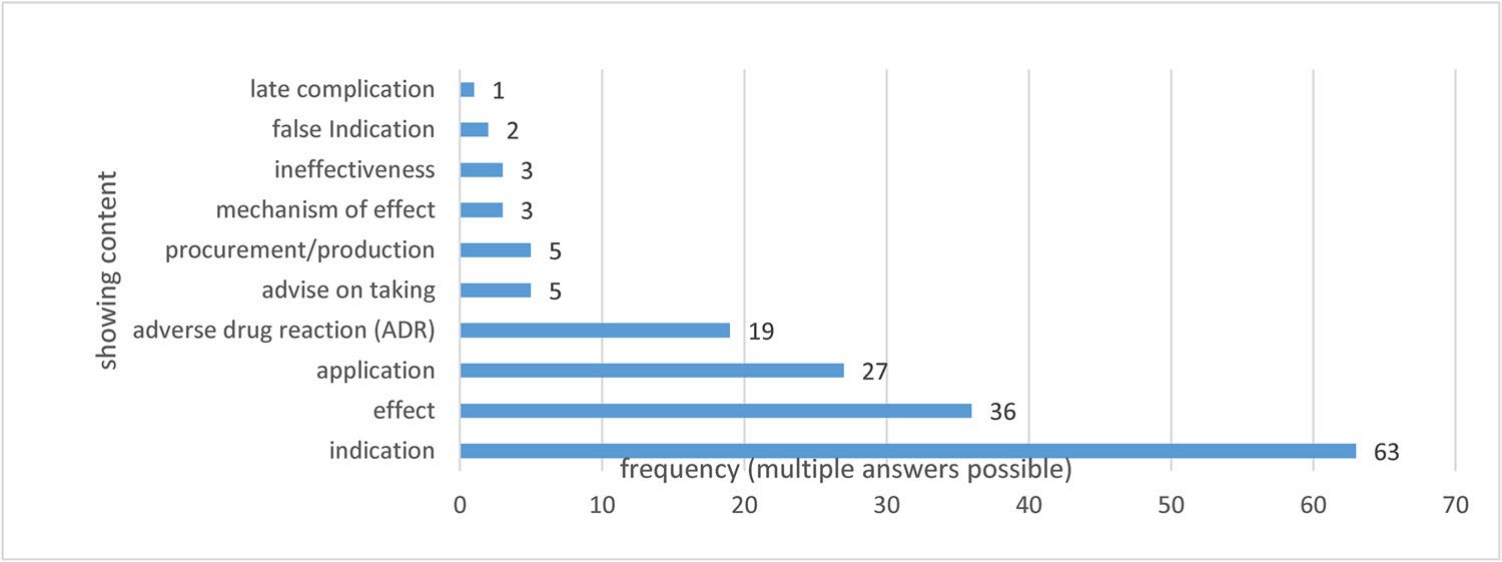
Presentation and content of drugs. Content-related discussion regarding the active pharmacological substances depicted in “Tatort” from 2019 to 2021 in absolute figures shown as a bar chart. Multiple presentations occurred in some cases

Figure 2 shows the presentation of active pharmacological ingredients of all episodes. Multiple presentations occurred. The substance itself was shown most frequently as a tablet or as a liquid in a syringe (41%), followed by naming in dialogues (33%). In 26%, the depiction of the effect was shown cinematically. Packages of medication were shown in 22% of the cases (some of them were created especially for the series, so they were fictional). The production or procurement of preparations was shown less frequently (5%). In episode 1162, the plant red foxglove was briefly shown, cryptically referring to cardiac glycoside preparations. Historically, in some countries such as Germany, cardiac glycosides had been often used in the treatment of heart failure. But internationally, cardiac glycosides are rarely used nowadays because of their toxicity. One of the adverse drug reactions of cardiac glycosides is sedation. (Seifert 2019a). Consequently, poisoning with this drug could have been presented in this episode. At the same time, red foxglove is used in the teaching of German homoeopathy for problems falling asleep and sleeping through the night (without medical evidence) (Herfurth 2018).

Looking to the content, the drug indication was identified most frequently (68%). In addition, the effect (39%) was shown, and adverse drug reactions were mentioned less frequently (21%). The application of active pharmacological substances was also shown (29%). Proportionally, oral administration was most frequently presented (44%); intravenous and intramuscular (11% each) as well as subcutaneous administration (7%) together were on par with inhalation of the active pharmacological substance: a cloth was hold in front of the patient, using an atomiser or using nasal cannula (19%). The tablet was crushed by the consumer and then dissolved in coffee or sniffed through the nose in two episodes (7%). There were also useful instructions for use, which mainly referred to fitness for duty and driving ability or limits for taking drugs (5%). The production or the possibility of procuring drugs was also discussed in “Tatort” (5%). One episode dealt with late complications (1%). The alleged indication was presented in the case of homoeopathic remedies, but always with the addition of ineffectiveness (2%).

As Ellerbeck and Seifert (2022) showed for poisons presented in the “Tatort” from 1974 to 2022, the mechanism of action of the active pharmacological substances was not shown very often: while it was reported for 12% for poisonings, it was only reported for 3% for drugs.

**Fig. 3:**
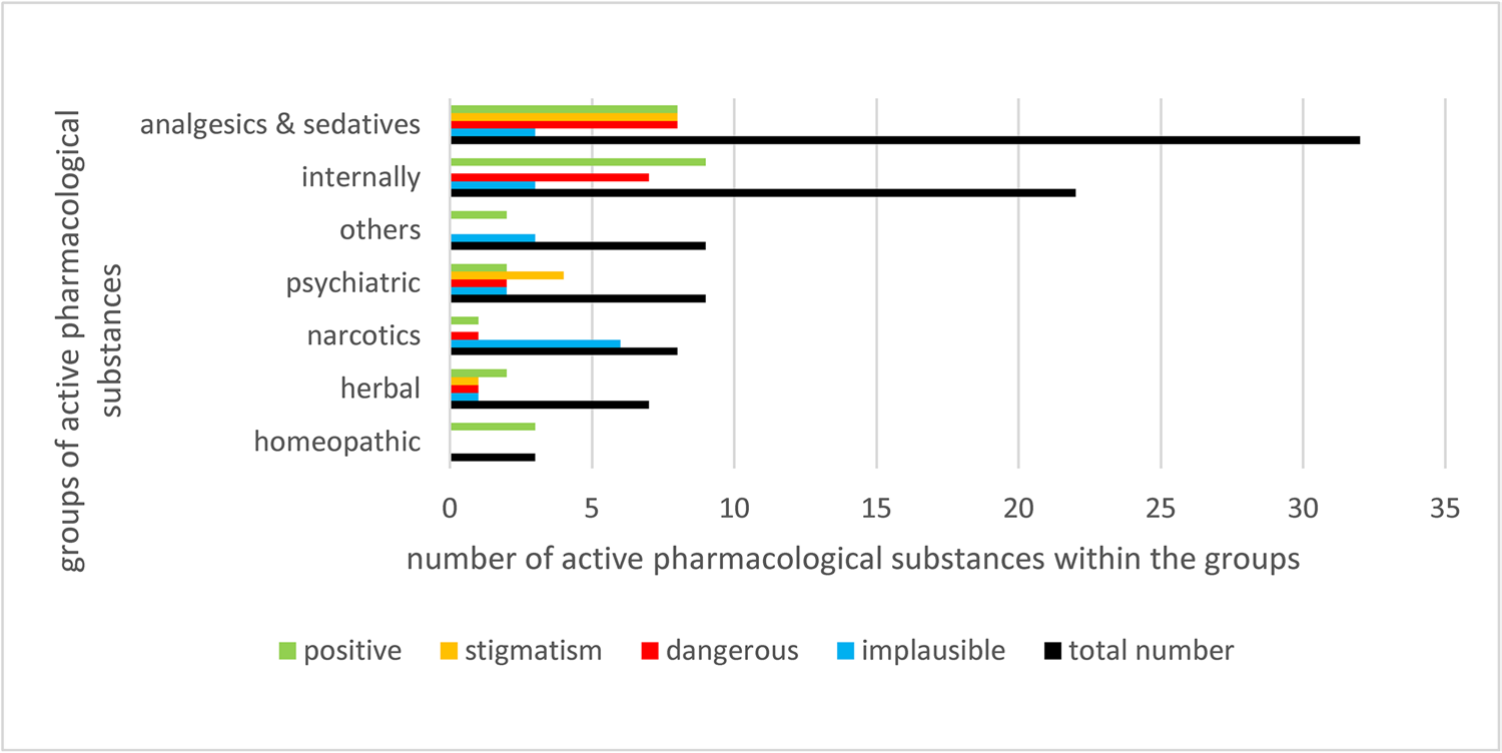
Comparison of individual drug groups (2019–2021). The individual drug groups mentioned in the crime scene are shown as a bar chart in relation to the frequency of implausible, dangerous, stigmatising and positive representations. Muscle relaxants, local anaesthetics and antidotes are summarised in the group “others”

Regarding the individual active pharmacological substances in every episode, these were checked for various parameters: If the presentation or passages of the presentations are implausible, if the viewers are informed about the active pharmacological substances in a benefit-oriented way (more than just presented correctly), if there is a possibility of imitation or abuse by viewer and if there is a risk of stigmatisation. Figure 3 shows the individual groups of active pharmacological substances in relation to the various parameters.

### Plausibility

Table 2 lists the active pharmacological substances that were implausibly presented. The majority of the depictions and uses of active ingredients in “Tatort” were plausible (82%); in most cases, there were only minor inaccuracies, which were presumably due to dramaturgy: 41% of the active ingredients were intended to render victims unconscious in a fast way. Chloroform (episodes 1113, 1129, 1149), which is no longer used in modern medicine, and other unknown agents (episodes 1051, 1141, 1171) worked quickly and reliably within a few seconds, and their depth of anaesthesia could be controlled well without monitoring; victims could be awakened immediately without an overhang. Chloroform does indeed have a sedative effect. However, this requires several deep breaths with a well-sealed mask enclosing the mouth and nose. Chloroform has a minimal alveolar concentration of 0.77 with a blood-gas coefficient of 8. Compared to sevoflurane, one breath is usually not enough to sedate with this agent. Furthermore, if unconsciousness were successful, the person would probably need to be ventilated, preferably secured by an endotracheal tube. In addition, circulation must be monitored, as the gas has a circulatory depressant effect (Solt et al. 2005). In the named episodes, effects on circulation and respiration were omitted, no airway management was needed.

**Table 2:** List of episodes with implausible depiction of pharmacologically active substances

Active substance (episode)	Content	Fact check
**Misinformation/false application**		
Cortisone (1155)	During a medical intervention of an anaphylactic shock, cortisone is made to appear to be the first-line agent	Anaphylactic shock is a common emergency that can happen to any doctor: the immediate application of adrenaline is indicated (intravenously or intramuscularly via autoinjector); “cortisone” (prednisolone) should also be given, but the onset of action is delayed: the membrane-stabilising effect appears later; H_1_R antagonists only help effectively against itching and urticaria, but not sufficiently in the case of oedematous swellings in the facial area. (Seifert 2019b)
Oxygen (1140)	An elderly person in a wheelchair is getting oxygen therapy via oxygen goggles. He gets severe shortness of breath and is in danger of suffocating because of squeezing the oxygen supply. (minute 57)	Oxygen therapy via goggles enables a supply of approx. 2 l/min which saturates the residual volume. Even when the oxygen is turned off directly, there is still a reserve capacity, and there is no (immediate) threat of suffocation. (Brunton et al. 2017h)
“Flumazenil” and caffeine (1092)	Forensic doctor explains that there is no antidote for benzodiazepines, but that caffeine can neutralise the effect (minute 50)	Flumazenil (antidote) binds to the benzodiazepine receptor at the allosteric binding site of the ionotropic GABA_A_ receptor that activates a chloride channel and is administered as an antidote in benzodiazepine overdose (Brunton et al. 2017d); caffeine is the most widely used psychoactive drug in the world; adenosine causes mild sedation when it binds to the adenosine receptor which can be antagonised by caffeine. (Brunton et al. 2017m)
Analgesics, unspecified (1081)	Commissioner is treated with strong painkillers, but no improvement (minute 5); she is symptom-free after esoteric Reiki application without physically contact (minute 56, 64)	It seems like medicine would not help in this case. Pain medication works for back pain, but exercise is important if red flags are excluded. (Hayden et al. 2005)
**Sedation**		
Digitalis (1162)	The plant red foxglove is briefly shown in a short cut while the anti-hero injects something into the upper arm of an awake person, who goes limp and falls unconscious (minute 79); the anti-hero obviously injects an antidote, the person wakes up directly, the anti-hero says that the drug always causes his wife to snore heavily and that it works very quickly (minute 80)	The cardiac glycoside digoxin is obtained from the poison of the foxglove; it inhibits the Na^+^/K^+^-ATPase. However, fatigue and sedation are only ADR (Seifert 2019a), so it is implausible that the toxic effect on the heart would have prevailed. Caution: in homoeopathy, red foxglove is also used as a sleep medication (Editors of globuli, 2012). https://www.globuli.de/einzelmittel/globuli-von-d-bis-f/digitalis/ (last accessed on 19.10.2022)
Digitalis antitoxin (1162)	Woman is injected with unknown drug, later she wakes up (minute 75); previously probably poisoned with digitalis preparation (see above)	On the hypothesis that it could be digitalis/cardiac glycosides, the antidote could be digitalis antitoxin which consists of digoxin antibody fragments from sheep. It is not actually used for unconsciousness, but in the event of cardiac arrhythmias. (Editors of Medical dictionary, 2015). https://medical-dictionary.thefreedictionary.com/Digitalis+antitoxin (last accessed on 16.10.2022)
Anaesthetic, unspecified (1171)	Kidnap victim is anaesthetised with intramuscular injection (minute 3)	This scene suggests good controllability without dangers and a false immediate impact. No monitoring or secured airway used; needle shown was too short. (Brunton et al. 2017i)
Anaesthetic, unspecified (1051)	Drug injected into the throat via fast-inject-pen immediately causes unconsciousness (minute 44)	Stereotype presentation: in case of unconsciousness, there is a high risk of overdose without control of respiratory and circulatory parameters. The active pharmacological substance is unclear, possibly ketamine (Brunton et al. 2017i)
Gas from cartridge, not further titled (1141)	Commissioner is attacked from behind, gets a mask, and very quickly becomes unconscious, although the mask is only on his mouth without the nose (minute 85); afterwards, he can easily be woken up	False representation of good controllability of the drug, no ADR or danger with no monitoring. There is usually a drop in arterial blood pressure up to circulatory depression; volatile gases are probably represented here: sevoflurane (in contrast to isoflurane) has a rapid onset of action, is also used for induction of anaesthesia (especially by children), concentrations of 2–4% are already sufficient due to the low solubility in blood and other tissues, but only a single breath as shown is insufficient for effect of unconscious. (Brunton et al. 2017j)
Chloroform (1113 min 10), 1129 min 25), 1149 min 70))	Victim becomes unconscious after first breath on a wet tissue; adverse effects or monitoring of anaesthesia are not shown	Implausible, unconscious requires several breaths to inhale enough of the active pharmacological substance. Chloroform has a minimal alveolar concentration of 0.77 with a blood-gas coefficient of 8. Compared with sevoflurane, one breath is usually not enough for effect. They use also only an impregnated cloth and not a tight-fitting mask; even with successful unconsciousness, the person would probably have to be ventilated and should be preferably intubated. (Solt et al. 2005)
Local anaesthesia, unspecified (1171)	For finger amputation, a local anaesthetic is applied to the wrist palmar radial. The person feels no pain in case of amputation (minute 55)	Active ingredient unknown, but erroneously direct onset of action after injection. Even the aminoamide prilocaine (Xylonest) or mepivacaine act as sodium channel blockers only after a delay. (Brunton et al. 2017k)
Glucose 40% (G40) (1143)	Application through clothing (minute 74), application with draw-up cannula into the three-way valve	Application should be made directly into the skin; application into the three-way valve is not possible due to lack of connection. (RKI 2011)
Glucose 40% (G40) (1114)	Application of G40 infusion in a peripheral venous (minute 68)	Application of G40 peripheral into venous is only allowed under running infusion due to venous irritation, unless there is an emergency like coma because of hypoglycaemia. (Fachinformation, 2007 (Summary of Product Characteristics) of B-Braun® on Glucose 40%)
**Psychiatric**		
Antidepressants (1090)	Clinic director suggests that you can identify people taking antidepressants by view (minute 17); declares that one year of LSD would cure his disease (minute 72); he is the anti-hero (minute 78)	Taking drugs against depression is not visible on the outside. The disease is presented as if it cannot really be treated with conventional medicine. One must resort to other active substances that are banned in Germany. There are many proven effective agents available for schizophrenia. (Härter et al. 2008)
“Medicine” (psychiatric) (1168)	One bigger theme of this episode is “mental disorder” which highlights psychosis. Medication does not work for a young patient (minutes 25, 51, 72)	This episode gives the impression that psychiatric drugs impair psychic abilities; patient has unexplained abilities in the sense of a premonition. No clinical improvement from psychiatric treatment is shown. (Härter et al. 2008)
“Benzos” (1149)	Monotherapy schizophrenia with “benzos”: looking at medical records, “benzodiazepines” are the only drugs listed for paranoid schizophrenia and alcohol addiction (minute 33)	Benzodiazepines are used in the treatment of schizophrenia only in addition to sedation, but not as monotherapy. (Brunton et al. 2017e)

The brief insertion of red foxglove in episode 1162 may have alluded to cardiac glycosides: in this case, the perpetrator used it successfully and without complication for sedation, contrary to reality. The cardiac glycoside digitalis is obtained from the poison of the red foxglove. In this process, the Na^+^/K^+^-ATPase is inhibited. However, the drowsiness and sedation that is exploited in this episode is only an undesirable drug effect that can occasionally occur. Because of the primary cardiac effect, an overdose that would cause sedation would probably lead to heart failure beforehand (Seifert 2019a).

Psychiatric drugs were presented in a blurred way with regard to therapy and adverse drug reaction (episodes 1090, 1149, 1168). The end of episodes 1090 and 1168 has the potential to raise the question in viewers to what extent psychiatric illnesses should be treated and whether active pharmacological substances approved in Germany are suitable for this at all.

In episode 1092, the forensic doctor is not aware of the antidote flumazenil for benzodiazepine overdose; he mistakenly advises taking caffeine, which he says sufficiently counteracts sedation. Caffeine is the most commonly used psychoactive drug in the world. The endogenous substance adenosine causes mild sedation when it binds to the adenosine receptor. This process is antagonised by caffeine. Contrary to what the Tatort-episode suggests, caffeine does not interfere with the mechanism of action of benzodiazepines and thus does not antagonise their effect (Brunton et al. 2017m).

In episode 1140, closing the oxygen mask closed for a short time leads to a state of threatened asphyxiation (Brunton et al. 2017h).

Contrary to the current guidelines, episode 1155 gives the impression that “cortisone” is the first drug of choice for the treatment of anaphylactic shock (Seifert 2019b).

Often, a sedative was injected in psychologically decompensated patients; a possible oral administration (episodes 1132, 1164, 1167) and crisis intervention through conversation was not considered beforehand (episodes 1122, 1188) (Table 3). In general, the use of benzodiazepines in acute traumatic situations is controversial, as these drugs influence episodic memory. It is currently being discussed whether this procedure promotes the tendency to develop post-traumatic stress disorder at a later stage (Klein 2008).

**Table 3:** Summary of the use of tranquillisers and other psychological interventions in mental crises

Tranquilliser (1088): Directly taking a tablet instead of trying to talk first, seems more dramatic in the scene (minute 15) Sedative (1122): Cleaning lady who has found the dead body is handed a tablet by the psychology intervention team (probably not a doctor, only they are allowed to give drugs) with the words: “Please take this, you need it now”. She then knocks the blue can out of her hand (minute 8); no try for a conversation by person, tablet could be offered but should not be forced on her Sedative injection (1132): Patient with borderline personality disorder wants to hurt herself with razor blades in a closed psychiatric ward. She is restrained in bed, calms down quickly after intramuscular injection (minute 10) (diazepam?). If necessary, a conversation should be first to calm down; a tablet should be offered orally Sedative injection (1164): Patient could also have taken the drug orally. (minute 33) Sedative injection (1167): Patient could also have taken the drug orally. (minute 40)	

The mode of action of the drugs in the Tatort episodes studied from 2019 to 2021 is even less frequently explained than for poisons from 1974 to 2022 (Ellerbeck, Seifert 2022), perhaps because the poisons were more often in focus of the action than drugs. In addition, according to Ellerbeck and Seifert 2022, symptoms were shown in 87% of the poisoning cases, whereas this was only the case in 39% of the drugs studied here. It should be noted that the work of Ellerbeck and Seifert deals exclusively with poisonings in episodes of “Tatort” from the years 1974–2021 in the sense of a longitudinal study.

### Educating the viewers

Table 4 lists the active pharmacological ingredients for which the viewers were profitably educated beyond the correct presentation which occurred in 29% of the cases. Illnesses can affect anyone, even an altruistic doctor was suffering from depression (episode 1181) or a trained commissioner from arterial hypertension (episode 1167) who were treated by using medication. It was pointed out that therapies can also be done without drugs, e.g., through better sleep hygiene for problems falling asleep and staying asleep (episode 1161) (DGSM S3-Guideline 2017) or a lifestyle change in arterial hypertension (episode 1167) (Badermann et al. 2020).

**Table 4:** Pharmacologically active substances for which the viewers were profitably educated beyond the correct presentation

Active substance (episode)	Content	Evaluation
**Education for common diseases**		
Antihypertensive (1167)	Commissioner explains when taking antihypertensive medication that with practising sports she might no longer need the medication (minute 18)	Education about widespread disease. Sport or lifestyle change in general as a potential way of not being dependent on tablets, good education and role model function by commissioner for the viewers. (Badermann 2020)
Analgesics, unspecified (1141)	Sport is good for back pain (minute 85)	Therapy advice: Exercise, not only or even exclusively analgesics are necessary for pain relief. (Hayden et al. 2005)
Sleeping pills (1161)	Commissioner suffers from insomnia, should first try sleep hygiene before drug therapy (minute 23); even illegal sleeping pills do not help (minute 68)	Doctor explains that medication is not useful here, person should first try sleep hygiene; the illegal substance does not really help her either; in this case, stress reduction is very important. (DGSM S3-Guideline 2017)
Antibiotic, antibacterial drug (1082)	Note of regular intake of antibiotic therapy (minute 45)	Education that antibacterial drug should not be discontinued earlier than prescribed. (U.S. Department of Health & Human Services 2021)
Antibiotic, antibacterial drug (1141)	Antibiotic as a life-saving drug by bacterial infections, placebo and homoeopathy with no effect (minute 44)	The importance of antibacterial drugs for today’s society is presented. Meningitis can also develop from a harmless bacterial infection which is shown in this episode. The ineffectiveness of homoeopathy is presented. (John Hopkins Medicine 2020)
Alpha-latrotoxin (1141)	In homoeopathy alpha-latrotoxin is used for arterial hypertension. Forensic doctor explains that it is a nerve poison and attests no effect except for the manufacturers regarding finances, calls it a placebo (minute 33)	Takes a good look at the subject of “homoeopathy”, homoeopathic efficacy is compared with placebo. (Michaelsen et al. 2017)
Homoeopathy (1141)	Child dies because he was treated with homoeopathy instead of antibacterial drugs (minutes 40, 44)	Takes a critical look at the subject, shows consequences and dangers of using homoeopathic preparations instead of antibiotics. The episode explains supposed success due to the placebo effect and presents superiority of modern evidence-based medicine. (Michaelsen et al. 2017)
Homoeopathic pill (1149)	Commissioner compares effectivity of placebo with effectivity of homoeopathy (minute 44)	Commissioner explains that homoeopathic preparations are just placebos and that there is no effect that comes from the preparation itself. (Michaelsen et al. 2017)
**Accidental poisoning**		
Sleeping pills, fictitious name (1093)	Woman accidentally poisons herself after her husband wanted to take his own life with an overdose of pills and leaves the glass of water with dissolved sleeping pills in the living room (minute 74, 76, 89)	Accidental poisoning is presented, which is very common in reality. Fictitious name of sleeping pills (Noctoporal) without possibility of inference. (Ellerbeck and Seifert 2022)
**Awareness of danger**		
“Rape-Drugs” (1159, 1183)	Rape drugs (“K.O.-Tropfen”) named without composition. Effect shown and how to identify them	Educating the public about the existence of rape-drugs without naming the exact active ingredient. Episodes showing ADRs up to death, showing the effect without giving instructions on how to obtain or produce it. (Schwartz 2000)
**Education about emergency pens in case of poisoning**		
Naloxone (1119)	In case of heroin overdose, use of naloxone is necessary. Episode shows also subcutaneous application (minute 7), followed by treatment in hospital (minutes 11, 14)	Education about existence of antidote. Correct application is shown; it is made clear that further treatment in hospital is necessary. (Brunton et al. 2017f)
Naloxone (1160)	Street worker explains that naloxone is the antidote for heroin overdose. It is available in the emergency kit (minute 42)	Mention that there is emergency medicine against heroin. (Brunton et al. 2017f)
Glucose 40% (1143)	Injection with a glucose-fast-pen for insulin poisoning is showing (minute 74)	Viewers are shown utilisation of a fast-pen in emergency. (Seifert 2019f)
**Common drug**		
Amiodarone (1095)	Person getting amiodarone against cardiac arrhythmia; this drug does not necessarily save life, because death is also possible by fate, explained by the doctor (minute 13), withheld the medication on a longer period had led to ventricular tachycardia and sudden cardiac arrest (minute 18)	Possibility is explained that fateful death is possible despite perfect medication; correct pathophysiology explained education about common disease; importance of continuous taking medication. [Brunton et al. 2017o)
**Information about therapy by common cold**		
Zinko Gold, fictitious preparation (1104)	No need for seeing a doctor for a common flu infection, uses vitamin preparations	Benefit is controversial, but taking vitamins seems to support the immune system; behaviour for simple colds is shown (do not contact a doctor). (Pecora et al. 2020)
Vitamins (1106)	Common cold disease, just consulting doctor if fever, told to rest as therapy (minute 9, 30)	Correct behaviour in the case of a common cold; in the case of fever, take an additional antipyretic and consult a doctor: rest and use over-the-counter preparations, drink plenty of fluids as a sensible therapy. (Pecora et al. 2020)
**Information about taking medication**		
Lorazepam (1180)	Commissioner explains that taking the active pharmacological substance would not allow service on the weapon (minute 37)	Indication of unfitness for duty/incapacity to drive if taken. (Halbe 2018)
**Mode of action**		
“Psychotropic drugs” (1132)	Psychiatrist explains that there are different psychotropic drugs for different forms of psychiatry illness. One works better than the other depending on the person (minute 60)	Education that there are different drugs for schizophrenia. (AWMF S3-Guideline 2019)
**Illness can affect anyone**		
Diazepam (1181)	Person (altruistic doctor) kept depression secret, took diazepam against disease (minute 23)	The disease can affect everyone in society, but you do not necessarily see it in patients. (Federal Ministry of Health Germany 2022)
Antidepressants (1181)	Person (altruistic doctor) kept depression secret, took antidepressants against disease (minute 23)	The disease can affect everyone in society, but you do not necessarily see it on patients. (Federal Ministry of Health Germany 2022)
Antihypertensive medication (1167) (double nomination)	Commissioner, who appears athletic and slim, takes antihypertensive medication (minute 87)	Commissioner as a normal person takes blood pressure tablets, reflects social average well. (Schoppe 2020)
**Significance of taking medication**		
Heart tablet (glyceryl nitrate?) 1158	Older woman has a “heart attack”, looks for her medicine in her handbag, and does not find it and dies (minute 2)	Bystander is made aware of a cardiovascular emergencyt; significance of emergency medication is shown. (Seifert 2019d)
Heart medication for heart rhythm disorder (1165)	Fall of an elderly lady as a result of exchanging regular medication (minute 31)	Education on the importance of taking the right medicines regularly. (Brunton et al. 2017n)
**Interaction**		
Antidepressants, unspecified (1181)	Doctor points out dangerous interaction when taking antidepressants and amphetamines together (minute 35)	Dangerousness of interaction, drug interaction is addressed. (Deutsche Hauptstelle für Suchtfragen e.V. 2022)
“Benzos” (1149)	Never taking benzodiazepines in combination with alcohol (minute 18)	Never take the drugs together with alcohol; benzodiazepines as a supportive agent in withdrawal, interaction is addressed as apnoea. (Brunton et al. 2017l)
**Representation of poisoning without imitation, but still plausible**		
Cardiac glycoside (11,622)	Plant red foxglove is briefly faded in which gives the guess for viewers, that the used medication could be cardiac glycoside (minute 80)	No obvious presentation of the active ingredient: if the medication is really cardiac glycosides, they are cryptically identifiable by the presentation for an expert audience, whereas the uninformed viewer remains clueless. Due to the presentation of a picture, it is also not easy to research for it on the Internet. (Seifert 2019a)
Muscle relaxant (1136)	Man is stunned with relaxants and caught deathly by a train (minutes 2, 5)	The risk of imitation is minimised here, as no specific drug is mentioned. However, the effects and ADRs described in the case of overdose are plausible for various preparations in this classification. (Brunton et al. 2017a)

The correct intake was pointed out for antibacterial drugs (episodes 1082, 1141): regarding the duration of taking antibacterial therapy, it should always be taken according to the doctor’s recommendation. Otherwise, taking the medication for too short or too long can promote resistance (U.S. Department of Health & Human Services 2021).

The same applied to the presentation of “heart medication” (episode 1165), and there was also reference to unfitness for duty as a policeman when taking lorazepam (episode 1180), whereas in episode 1092, contrary to current case law in Germany, no strict driving ban was imposed by a forensic doctor prescribing lorazepam. Driving ability is impaired by taking benzodiazepines. According to German law, for example, it is no longer permissible to drive a vehicle on German roads under this effect. A doctor must point out the inability to drive (Halbe 2018).

Normal common cold infections were never treated with antibacterial therapy, but at most with vitamin-mineral preparations (Pecora et al. 2020). A doctor was not consulted for this if there were no problems like fever (episodes 1104, 1106, 1141).

The existence and use of antidotes like naloxone and flumazenil was discussed (episodes 1092, 1119, 1143, 1160). Naloxone is used as an antidote for opioid overdose like heroin. A street worker explains these circumstances in episode 1160. In episode 1190, the intramuscular application of an antidote is shown. Flumazenil is administered as an antidote in benzodiazepine overdose (like diazepam), binding to the benzodiazepine receptor at the allosteric binding site of the ionotropic GABA_A_ receptor that activates a chloride channel (Brunton et al. 2017f). The scenes educate viewers on the use of emergency pens and antidotes in case of poisoning.

The non-existent pharmacological efficacy of homoeopathy was also shown (Michalsen et al. 2017) as well as the dangers of using homoeopathic medication instead of antibacterial drugs (episodes 1141, 1095). Meningitis can also develop from a harmless bacterial infection which is shown in episode 1141. Antibiotics (antibacterial drugs) inhibit bacterial metabolism, preventing the spread of disease. (Martindale et al. 1989). Late or omitted administration of antibacterial drugs can lead to deadly complications, as shown in this episode.

### Derivable dangers

Table 5 lists the active pharmacological substances where dangers to viewers can be inferred. In Tatort, ways are presented to kill or harm other persons. In 20% of the “Tatort” episodes, dangers for the public could be identified.

**Table 5:** Derivable dangers through the representation of pharmacologically active substances in the individual crime scene episodes

Active substance (episode)	Content	Evaluation
**Treatment**		
Cortisone (1155)	In the context of anaphylactic shock, the impression is created that “cortisone” (correct designation is prednisolone) is the first-line active pharmacological substance	Danger of false imitation. Skilled professional personnel are also misinformed. The threshold to use adrenaline is high due to the potential side effects; but in this case, it is the only active substance that saves the patient lives. Prednisolone only takes effect with a delay. Cortisone is a very widely used jargon term for glucocorticoids. Cortisone is the inactive precursor of cortisol (hydrocortisone) and has no place in the therapy of anaphylactic shock. (Seifert 2019b)
**Procurement**		
Ritalin® (methylphenidate) (1177)	A schoolgirl explains that, like many people, she takes the drug to improve her performance (minute 18). The movie franchise positively portrays the improvement in performance due to the drug (minute 46). Her cousin working at a special school would collect the drugs because she did not approve of taking them (minute 19)	The episode presents consumption as a legitimate method of enhancing performance in our society. Glorification of the effect through special camera settings positive portrayal of the drug mention the risk by takings. Methylphenidate is an indirect dopamimetic (promotes the neuronal release of dopamine) with the result of increasing the ability to concentrate. Caveat: dependence problems and tachyphylaxis have not been addressed—at some point (due to the depletion of the vesicles), a performance kink occurs. At the same time, a way of procuring the drug and the possibility of earning money by fencing stolen goods are presented. (Seifert 2019c)
Ephedrine (1167)	Adding ephedrine into drink causes person to die while jogging (minute 3). Active ingredient is used to treat a common dog disease (urinary incontinence), can easily be obtained from veterinarian (minute 61)	Viewers get instructions for obtaining the active pharmacological substance, which in this case has a lethal effect. Ephedrine is an indirect sympathomimetic. Dispensing rights actually lie with the veterinarian; an easy procurement is possible as shown in the episode; often, the animal does not even have to be brought along. (Editors of drugs.com 2017). In human medicine, ephedrine is no longer used due to more effective alternatives. (Gollakner 2019)
Insulin (1143)	Presentation of poisoning and killing with insulin (minutes 4, 61), explanation that it was difficult to prove (minute 39)	Instructions for almost undetected murder. Medicine is easily accessible because many people suffer from diabetes. Hypoglycaemia frequently leads to falls, which is one of the most common injuries in old age. Difficult to detect murder with insulin. Insulin, as the most important factor in diabetes mellitus, stimulates the uptake of glucose into the cell and glycogen synthesis and inhibits lipolysis, thus actively lowering the blood glucose concentration. (Seifert 2019e) There is also an application error within the episode: insulin is injected via pull-up cannula into a three-way valve
Fentanyl (1160)	Street worker explains to the commissioners that used fentanyl patches can be found in household rubbish. They are boiled out and injected or chewed (minute 43). A geriatric nurse abuses this as a nasal spray to sleep, getting a “nothing matters” feeling (minute 65)	Instructions for obtaining an anaesthetic: Fentanyl (opioid analgesic; MOR agonist) as a patch is used for pain patients. (Brunton et al. 2017g) Disposal in household waste is possible; patches may only be used for a maximum of 3 days, after which up to 50% of the active ingredient is still present, but the patch delivers no longer a constant dose. (Editors of DAZ 2018) Fentanyl as a nasal spray is very addictive. (Editors of medline-plus 2020)
Fentanyl (1111)	A drug informant explains to the commissioners that fentanyl can be boiled out of patches, which have to be changed after 3 days after use (minute 32)	Instructions for obtaining an anaesthetic (opioid analgesic; MOR agonist). (Editors of DAZ 2018)
Presumably fentanyl (1096)	Forensic doctor explains to the police that fentanyl patches contain an active ingredient that “junkies” boil out or chew directly. A laboratory for boiling out the patches is shown; a hint is given on how to find instructions on the Internet. (minute 31)	Instructions for obtaining an anaesthetic (opioid analgesic; MOR agonist). (Editors of DAZ 2018)
**Risky consumption**		
Lorazepam (1092)	Forensic doctor prescribes commissioner “Lorazepam 1–0-1–0”, advises her to be careful when driving because of the fatiguing effect (minute 51)	Doctor is downplaying the strong sedative effect. Lorazepam belongs to the benzodiazepines and acts at the GABA-A receptor, thus having a sedative-hypnotic, muscle-relaxant and anxiety-relieving effect. It leads to amnesia and is anticonvulsant (Brunton et al. 2017d). In Germany, driving a motor vehicle under the influence of benzodiazepines is prohibited. The doctor must point this out and ensure that this is done if necessary. (Halbe 2018)
Headache tablet, unspecified (1129)	A tablet is taken when drinking heavily (minute 7); the next day another commissioner takes two headache tablets at once (for hangover) (minute 18)	It is not recommended to take analgesics while being drunk. Many sources explicitly warn against taking analgesics during alcohol consumption, as moderate interactions such as intestinal bleeding may occur. (Editors of drug.com 2022). In this case, the colleague even asks his partner to take two at once and thus probably overdoses
Analgesics, unspecified (1179)	After outpatient treatment of a gunshot wound, anti-hero drinks stronger alcohol at home (minute 60)	Antihero is probably under the effect of pain medication, see above. (Editors of drug.com 2022)
Antihypertensive (1130)	A commissioner dissolves antihypertensive tablet in coffee after a bad night (minute 43)	Tablets should not be changed in their dosage form. Take them always with water; coffee also increases blood pressure. [AOK 2021]
“Zinko Gold”, fictitious (1104)	In the context of a common cough, the commissioner takes several tablets of the herbal preparation without first reading the package carefully (minute 22)	The instructions on the package should be followed with every drug including herbal preparations. Uncontrolled swallowing of several tablets without counting is a risky behaviour in terms of provoking an overdose. (AOK 2021)
**Use of rape drugs**		
Benzodiazepine (1092)	Forensic doctor explains that the drug used is a benzodiazepine, which in the right dosage acts like a rape drug (K.O.Tropfen) (minute 50). An unconscious commissioner without respiratory monitoring is awakened with antidote after injection with benzodiazepines. (minute 71)	This episode shows an abuse to sedate people. Additionally, good controllability and high therapeutic breadth are suggested. In high doses, the drug has a hypnotic effect, associated with many ADRs such as respiratory depression. (Brunton et al. 2017c)
Rape drugs (K.O.-Tropfen) (gamma hydroxybutyric acid), 1159	Victim died of an overdose of rape drugs (K.O.-Tropfen), says the forensic expert. He calls the active ingredient gamma hydroxybutyric acid, which is like liquid ecstasy (minute 9)	This episode shows an abuse to sedate other people by exact naming of the substance as well as naming the colloquial name. Imitation is made easy. A rapid onset of symptoms is shown. Too high doses lead to respiratory depression and coma and even death. Abuse as a “date rape drug” is common in Germany. (Andresen et al. 2008)
**Changing medicines for killing**		
Amiodarone (1095)	Elderly man in need of care dies after nursing service switched amiodarone for a placebo over several days (minute 44)	Instructions for murder: changing of active pharmacological substances (in this case amiodarone). Amiodarone is used primarily for therapy-resistant ventricular tachycardia and atrial fibrillation. (Brunton et al. 2017o)
Heart medication (against cardiac arrhythmia) (1165)	Fall of an elderly lady is hypothetically attributed to changing her medication with caffeine (minutes 4, 31)	Instructions for murder: the exchange of drug to harm/kill people: active pharmacological substance is not named, but it could be amiodarone. (Brunton et al. 2017n). This idea is implausible, as amiodarone has a very long half-life. Only after several weeks does the concentration of active substance decrease. The person died because of other circumstances in this episode

Insulin was introduced as a difficult-to-detect murder agent in episode 1143, leading to death either directly because of hypoglycaemia or through accidents in the context of neurological deficits, including the depiction of easy subcutaneous application. Hypoglycaemia often causes falls, which are one of the most common injuries among elderly. Insulin, a key factor in diabetes mellitus, stimulates glucose uptake into human cells and glycogen synthesis and inhibits lipolysis, lowering the blood glucose concentration. (Seifert 2019e) Since diabetes is a widespread disease in Germany, accessibility to this medication is easy, the active substance is well known, and its effect is very potent (Seifert 2019e). There is also an application error within episode 1143: Insulin is injected via pull-up cannula into a three-way valve.

The extraction of narcotics such as fentanyl by boiling or sucking fentanyl patches was also discussed in detail in several episodes (Episode 1160, 1096, 1111). Fentanyl, a powerful synthetic opioid analgesic, as a patch is used for pain patients (Brunton et al. 2017g). Disposal in household waste is possible; patches may only be used for a maximum of 3 days, after which up to 50% of the active ingredient is still present, but the patch delivers no longer a constant dose (Editors of DAZ 2018). In episode 1160, a geriatric nurse uses fentanyl nasal spray as an easy way to fall asleep. This is particularly alarming because fentanyl in this form of application has a high potential for dependence (Editors of medline-plus 2020).

Episode 1167 used ephedrine for killing: ephedrine is used for urinary incontinence in dogs and is easy to obtain from the veterinarian who actually holds the dispensing right in Germany (§ 43 Abs. 4 und 5 Arzneimittelgesetz (Medicines Act) of Bundesrepublik Deutschland, current version from 1976).

Episode 1177 showed a schoolgirl under psychological pressure who, according to her own statement, was receiving methylphenidate through an employee of a special school. The employee had stolen the tablets from her schoolchildren in the morning to sell them abusively. Methylphenidate is an indirect dopamimetic, structurally related to amphetamine. Its effect is more strongly geared to mental than to motor activity, which is why it is often abused in relation to other drugs. The substance has its indications in ADHD and narcolepsy. (Brunton et al. 2017b). The effect of tachyphylaxis leads to a complete depletion of the vesicles when taken continuously, which causes a performance kink (Seifert 2019c).

Furthermore, the omission or substitution of permanent medication showed alternative possibilities of murder (episode 1095, 1165). Risky behaviour when taking active substances was also presented: taking analgesics in combination with alcohol against headaches or while having a hangover (Editors of drugs.com 2017) as well as swallowing several tablets at once without reading the package leaflet (episodes 1093, 1104, 1109, 1129, 1130, 1179). At the same time, the active ingredient of analgesics was often not explained in detail (episodes 1081, 1093, 1109, 1171). Episode 1155 at least suggested that “cortisone” (which may mean prednisolone) is the emergency drug of choice for an anaphylactic shock. Adrenalin, however, is considered a first-line medication due to its immediate onset of action. Only adrenalin is lifesaving in this case, whereas prednisolone has a membrane-stabilising effect only after a long delay. This effect supports in a later phase; likewise, H_1_R antagonists only help effectively against itching and urticaria, but not sufficiently against oedematous swellings in the facial region. This is a misrepresentation with consequences, especially remembering that anaphylactic reactions can affect any doctor at any discipline. Also, adrenaline with its large adverse drug reaction profile can generally have a deterrent effect to doctors (Seifert 2019b).

### Possibility of stigmatisation

Table 6 lists the active pharmacological substances for which there could be a risk of stigmatisation by viewers. Stigmatisation occurred with 16% of the active pharmacological substances shown in “Tatort”. Analgesics were denied their effect (episode 1081) or were declared as “pure chemistry” (episode 1096). Elsewhere, pharmacological therapies were demanded, and occupational therapy was considered useless (episode 1109). In modern pain therapy, occupational therapy is an important component of therapy for reducing pain. Its effectiveness has been proven in studies, especially for chronic pain (Nielsen 2021). Benzodiazepines were said to have an extremely addictive potential; moreover, the positive effects were not highlighted nor saying why it may be important to take these drugs (episode 1177). A similar presentation happened for “antidepressants” (no differentiation for the specific active substance was made), which were mainly portrayed negatively: a clinic director suggests that you can identify people taking antidepressants just by viewing the patients face (episode 1090) or the medication turned patients “into zombies” (episode 1104). Also, about “psychiatric drugs”, a doctor explained that pharmacological therapy had the power to make people mentally ill instead of alleviating the symptoms of the disease (episode 1132). This is particularly alarming because psychiatric diseases are also stigmatised in society, and the patient group is particularly vulnerable about adherence to medication. Adverse drug effects often initially overshadow the benefits at the beginning of therapy, which leads to an increased dropout rate (Cesková 2009). In episode 1177, Methylphenidate (Ritalin®) was presented on the one hand as a “miracle drug” for mental peak performance—for example, a schoolgirl succeeds in joining an orchestra, which is filmed with impressive camera settings and backed with virtuoso music—and on the other hand, the rejection of the drug by an employee at a special school, here appearing as an eminence for the viewer, who collects the methylphenidate in the school and then sells it for profit. “Tatort” has also investigated the topic homoeopathy in episode 1141, which is reflected very critically. The danger in case of using homoeopathic medication as a monotherapy is shown: an infant dies because of not treating streptococcal meningitis with antibacterial drugs. It should be noted, however, that in this episode, there was no clear separation from herbal preparations—a circumstance that the homoeopathy industry in Germany also uses for profit (editors of aponet.de 2022).

**Table 6:** Stigmatisation of pharmacologically active substances in Tatort episodes from 2019 to 2021

Active substance (episode)	Content	Evaluation
**Heroisation**		
Ritalin® (methylphenidate)	Downplaying of the adverse effects of Ritalin®; taking it is legitimate in high-performance society (minute 18). A special schoolteacher denies the therapy with methylphenidate and even collects it from her pupils (minute 19). A child plays virtuously under the effect of methylphenidate, clearly visualised by special pictures and style of the camera (minute 46)	The active ingredient is stylised as a miracle drug with which every child can achieve everything. You have a trust in special schoolteachers. In this scene, she refuses to take the medicine. There is evidence for the effectiveness of methylphenidate in ADHD. (Brunton et al. 2017b)
**Inferiority of medication**		
Analgesics, unspecified (1081)	Commissioner has back pain, is already taking strong pain medication without success (minute 20), Reiki without physical contact reduces the pain-level completely (minutes 56, 64)	Analgesics are presented as not useful in therapy against back pain. Also, movement and mobilisation are generally important for back pain. In this episode, a non-contact Reiki-application helps here, creating the impression that alternative medicine is superior to evidence-based medicine. (Nielson 2021)
Fentanyl (1096)	Commissioner rejects a painkiller with the words: “chemistry is bad for the liver” (minute 13). Commissioner smokes joint against pain (minute 57), Antihero also does not want analgesics because of liver toxicity, instead of this he wants a joint (“Tüte”) (minute 69)	Analgesics and other active substances are presented as bad and “poisonous”. Also, “herbal remedies” like marijuana should help better and should be healthier. As an authority figure for the viewers, the commissioner smokes joints because it might help against back pain (Hayden et al. 2005). It is not made clear that herbal remedies are also “chemicals”
**Showing just selected side effects**		
Antidepressants, fictitious preparation Dawex, not further described (1104)	Ingestion of antidepressants “turned [him] into a zombie” (minute 76)	Especially at the beginning of the pharmacological therapy, adverse effects predominate positive effects. The adherence is generally low; due to the illness, patients are more often distrustful. Also, psychiatric illnesses are generally stigmatised in our society (Cesková 2009). In addition, methamphetamines tend to cause “zombie”-conditions, but not antidepressants (very heterogeneous groups of active substances are hidden behind the name)
Antidepressants (1090)	Clinic director suggests that you can identify people taking antidepressants by view (minute 17)	This episode implies people taking antidepressants can be detected by a short view. As the disease depression is generally stigmatised, adherence falls even further. (Seifert 2019g)
“Psychotropic drugs” (1132)	A doctor says that healthy person therapied with psychiatric medication would become ill (minute 34)	Unsightly presentation especially for persons who are treated with psychiatry medication. For example, paranoid patients’ adherence could decline further. (Cesková 2009)
Diazepam (1177)	Doctor says that benzodiazepines are extremely addictive without explaining why they may be important in treatment (minute 54)	Only dangerousness is shown, not the benefit of taking the medication. The adherence could decline further in a generally already distrustful clientele. (Brunton et al. 2017c)
Benzodiazepines (1177)	When witness is asked after noticing that his girlfriend was taking “benzos”, he replied that she had seemed “unambitious” and had put on a “little weight” (minute 38)	The ADRs are elaborated, but the benefit of the drug is not presented. The adherence could decrease further in a generally already distrustful clientele. In addition, H_1_R antagonists tend to lead to weight gain, but not benzodiazepines. (Brunton et al. 2017c)
Analgesics, unspecified (1109)	Patient refuses “Ergo” after shoulder dislocation and thinks this “sucks”, demands pain medication, and takes it himself (minute 58)	Showing ineffectiveness of occupational therapy, only analgesics would help; taking tablets is more convenient than physical exercise or lifestyle change; attitude is further encouraged here by better effect. (Nielsen 2021)
Sedative (1141)	Commissioner must abort MRI examination due to claustrophobia. He compares the drug for sedation taking before the investigation with “gummy bears” (minute 85)	Episode shows ineffectiveness of therapy, adherence could be decrease. (Brunton et al. 2017c)
**Blurred boundaries**		
Vitamins (1141)	The episode deals critically with homoeopathy, in one scene you also see vitamins in the form of posters (minute 17)	This episode critically examines the homoeopathic scene, but here herbal remedies are presented. There is no sharp distinction between homoeopathy and herbal remedies (homoeopathy likes to present itself as herbal). (Müller 2019)
Herbal drops (1141)	Episode deals critically with homoeopathy; in one scene, you can identify a wooden cartridge with drops and illustrations of plants. (minute 17)	This episode critically examines the homoeopathic scene, but here herbal remedies are presented. There is no sharp distinction between homoeopathy and herbal remedies (homoeopathy likes to present itself as herbal). (Müller 2019)
**Other**		
Sedative syringe (1108)	Commissioner forces hostage-taker to give up: “… inject you in your bony butt” and threatens to send her to the “loony bin” (minute 63)	Statement suggests arbitrariness in giving psychiatric illness, an injection would make people compliant and label them as mentally ill. Here, it is meant to be funny. Meanwhile, midazolam, for example, would also rather be given nasally. (Brunton et al. 2017c)

Although the active ingredient of analgesics is named precisely in many episodes, in other episodes, the drug is simply referred to as “painkillers”. In these episodes, the active ingredients and their specific areas of use are not discussed in detail, nor is the profile examined more closely (episodes 1081, 1093, 1109, 1129, 1171, 1179).

## Limitations and conclusions

The crime series “Tatort” is fictional. Nevertheless, it takes on socially significant topics such as widespread diseases and generates an opinion among viewers about active pharmacological substances in medicine. At the same time, ARD® broadcasts not only entertainment but also “educational television”. Thus, it could be expected that other movie franchises in television and Internet without educational agenda make even more mistakes in relation to active pharmacological substances than ARD®. Moreover, it was often difficult to draw conclusions about the active ingredients because they were not explicitly named or shown.

The television-show “Tatort” teaches Germany and many other countries about active pharmacological substances. Doctors must be aware of this, especially when prescribing drugs: education about active ingredients is important because every patient is passively informed by watching movies and series. This information can be incorrect or questionable, which can massively limit their adherence—active reading of the package insert is not necessary to become unsettled. In depictions of drug abuse, there is a natural dilemma in films between providing information to protect the public and at the same time instructing people to commit a crime. However, Tatort, which also addresses common diseases such as back pain, hypertension or depression, shows corresponding medication therapy options and deals with topics such as antibacterial resistance and homoeopathy—but not always in the necessary depth and range which would be required. Especially active pharmacological substances that are used as analgesics or in psychiatry are particularly affected—in a peer group that already shows lower adherence (Cesková 2009).

Most of the mistakes were made in the stage of writing the script, which could have been easily avoided through proper medical advice or discussion. But the final movie still involves actors, directors, producers and film editors, as well as the “Freiwillige Selbstkontrolle der Filmwirtschaft” (FSK, an institute to control violence, sex and drugs in movies), which can rearrange or remove scenes.

To minimise imitation of dangerous behaviour like committing a murder, the series sometimes resorts to fictitious product names (10% of all names in the period shown) or makes cryptic references to ingredients, for example by using images of red foxglove, which is supposed to stand for cardiac glycosides.

In the future, it would be desirable to involve personnel with pharmacological expertise in the production of “Tatort”-episodes at the script writing stage to avoid stigmatisation and misinformation and at the same time provide education for the audience without encouraging abusive behaviour. “Tatort” script writers should take the opportunity and educate the audience on the mechanism of action of commonly used drugs. Understanding how drugs work can make a great contribution towards increasing drug therapy adherence and reducing stigmatisation of valuable drugs.

## Data Availability

A highly detailed pharmacological analysis of all Tatort episodes (source data) is available from the authors upon reasonable request.
